# Identification and Characterization of a Cytosolic Malate Dehydrogenase as a Potential Drug Target in *Echinococcus multilocularis*

**DOI:** 10.1021/acsomega.6c05466

**Published:** 2026-10-26

**Authors:** Pascal Zumstein, Laura Vetter, María Eugenia Ancarola, Trix Zumkehr, Britta Lundström-Stadelmann

**Affiliations:** † Institute of Parasitology, Department of Infectious Diseases and Pathobiology, 685610 University of Bern, Bern 3012, Switzerland; ‡ Graduate School for Cellular and Biomedical Sciences (GCB), University of Bern, Bern 3012, Switzerland; § Multidisciplinary Center for Infectious Diseases, University of Bern, Bern 3012, Switzerland

## Abstract

Alveolar echinococcosis, caused by the larval stage (metacestode) of *Echinococcus multilocularis*, is an emerging zoonotic disease with limited treatment options, highlighting the need for new therapeutic targets. Like other helminths, *E. multilocularis* relies on anaerobic metabolism via malate dismutation, a poorly characterized pathway. In this study, cytosolic malate dehydrogenase (cMDH), a key enzyme in this pathway, was investigated as a potential drug target. Bioinformatic analyses identified three cMDH homologues in *E. multilocularis*, all exhibiting canonical domain architecture and clustering with metazoan orthologs. Transcriptomic profiling revealed that EmuJ_000417100 (*Em*cMDH) is highly expressed in both *in vitro*-cultured metacestode vesicles and parasite lesions in mice, whereas other paralogs show minimal expression. Recombinant *Em*cMDH catalyzed the reversible conversion of malate and oxaloacetate, displaying typical cMDH kinetics with higher catalytic efficiency and lower *K*_m_ values for oxaloacetate (16.4 ± 3.2 μM) and NADH (11.5 ± 3.9 μM) compared to malate (0.21 ± 0.10 mM) and NAD^+^ (0.43 ± 0.11 mM), indicating a preference for oxaloacetate reduction. RNAi-mediated knockdown of *Em*cMDH impaired metacestode vesicle development, demonstrating its important role in parasite growth. The natural compound gossypol potently inhibited *Em*cMDH (IC_50_ = 0.25 ± 0.04 μM) with ∼80-fold selectivity over murine cMDH. As proof-of-concept, gossypol also reduced parasite viability (IC_50_ = 6.43 ± 0.66 μM), though cytotoxicity was observed in mammalian cells. While the known promiscuity of gossypol precludes attributing these effects solely to *Em*cMDH inhibition, these findings hint at *Em*cMDH as a functionally relevant metabolic enzyme and a potential drug target, providing a foundation for further validation and development of selective cMDH-targeted therapies.

## Graphical abstract

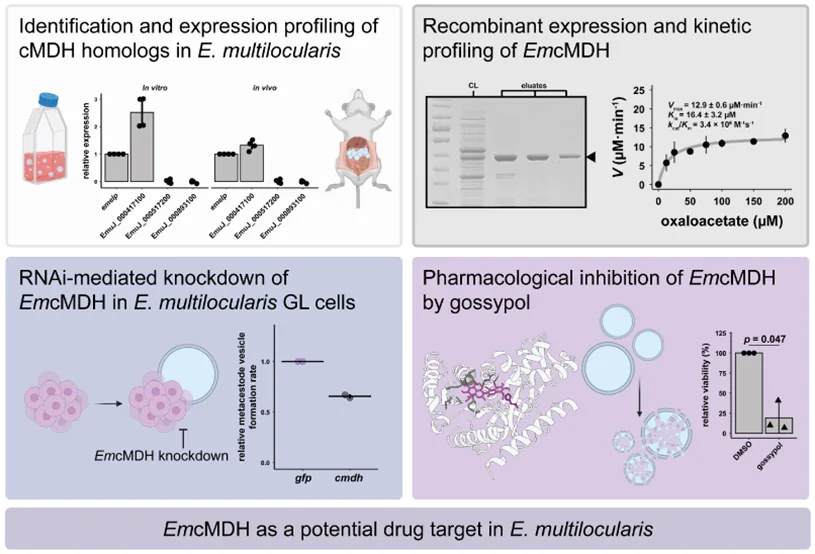

## Introduction

The larval metacestode stage of the fox tapeworm *Echinococcus multilocularis* is responsible for alveolar echinococcosis (AE), a severe, neglected zoonotic disease affecting humans as well as other mammals such as dogs and nonhuman primates.^1^ Infection occurs following ingestion of parasite eggs, which release oncospheres that typically migrate to the liver and develop into metacestodes, the disease-causing stage.^2^ Metacestodes are fluid-filled vesicles consisting of a complex wall structure composed of an outer carbohydrate-rich, acellular laminated layer, surrounding an underlying syncytial tegument layer, and an inner cellular germinal layer (GL).^3^ The GL comprises various cell types of which around 20–25% are undifferentiated stem cells that contribute to the high regenerative capacity of the parasite and they play a key role in disease progression.^4^ AE is characterized by invasive, tumor-like growth within tissues, and the parasite may also metastasize to secondary organs.^5^ Without treatment, the disease is fatal. Each year, about 10,489 new cases are reported worldwide,^1^ and the overall global burden has been estimated at 688,000 disability-adjusted life years.^6^ Although case numbers remain relatively low, incidence is increasing in Europe,^7^ and the parasite is emerging in new areas.^8^

The preferred AE treatment is surgical removal of the entire parasite mass, although this is feasible in only 20–50% of cases in countries with good access to advanced healthcare systems.^9^ Surgery is combined with long-term administration of benzimidazole (BMZ) drugs, such as albendazole or mebendazole.^10^ For patients who cannot undergo curative surgery, BMZ therapy remains the sole option. However, BMZs generally act parasitostatically, halting parasite growth rather than killing it, so relapse occurs if treatment is stopped.^11^ Consequently, patients often require lifelong daily medication.^10^ Prolonged use of BMZs leads to mild side effects in 54.5% of patients and 6.9% suffer from life-threatening side effects, particularly hepatotoxicity, which leads to discontinuation of drug treatment.^9^ Therefore, current BMZ therapy presents many drawbacks, and novel options are urgently needed.

Whole-organism-based drug screening assays have been developed for *E. multilocularis* and *E. granulosus* *sensu stricto*, enabling the establishment of a structured *in vitro* screening cascade. This includes objective assays such as damage-marker release and viability assays, which can be applied to both metacestodes and isolated GL cells to assess parasiticidal activity.^12,13^ However, whole-organism screening is resource-, labor-, and time-intensive.^14^ As an alternative, target-based drug screening enables high-throughput approaches at lower cost and facilitates hit identification and lead optimization, including the use of computer-aided drug design.^15^ Despite these advantages, it requires detailed knowledge of parasite biology and host–parasite interactions.

Unlike their mammalian hosts, many helminths rely on anaerobic pathways for ATP generation, particularly within the hypoxic niches they inhabit.^16^ A central feature of this metabolism is malate dismutation (MD) (Figure 1). MD is a fermentative pathway for the catabolism of carbohydrates: Phosphoenolpyruvate derived from glycolysis is converted to oxaloacetate via phosphoenolpyruvate carboxykinase. The generated oxaloacetate is subsequently reduced to malate by the cytosolic malate dehydrogenase (cMDH) and imported into mitochondria. In the mitochondrion, malate can be oxidized (a) or reduced (b). (a) In the oxidative branch, malate is oxidized to acetate which generates reducing equivalents in the form of NADH via the NAD-dependent malic enzyme and the pyruvate dehydrogenase complex and further leads to substrate-level phosphorylation via the action of acetate:succinate CoA-transferase and succinyl-CoA synthetase. (b) In the reductive branch, malate is reduced to fumarate via fumarase, which is then further reduced to the main end-product succinate. In this branch, fumarate acts as a final electron acceptor in an alternative electron transport chain, where complex I generates a proton motive force and complex II acts in reverse as a fumarate reductase rather than a succinate dehydrogenase, facilitated by an unusual hydrophobic electron carrier, rhodoquinone.^17^ The reductive branch is essential to maintain redox balance and oxygen-independent ATP generation via the alternative electron transport chain.^17^ cMDH represents a key enzyme involved in this pathway as it generates the majority of the malate that is reduced and oxidized in mitochondria.

**Figure 1:**
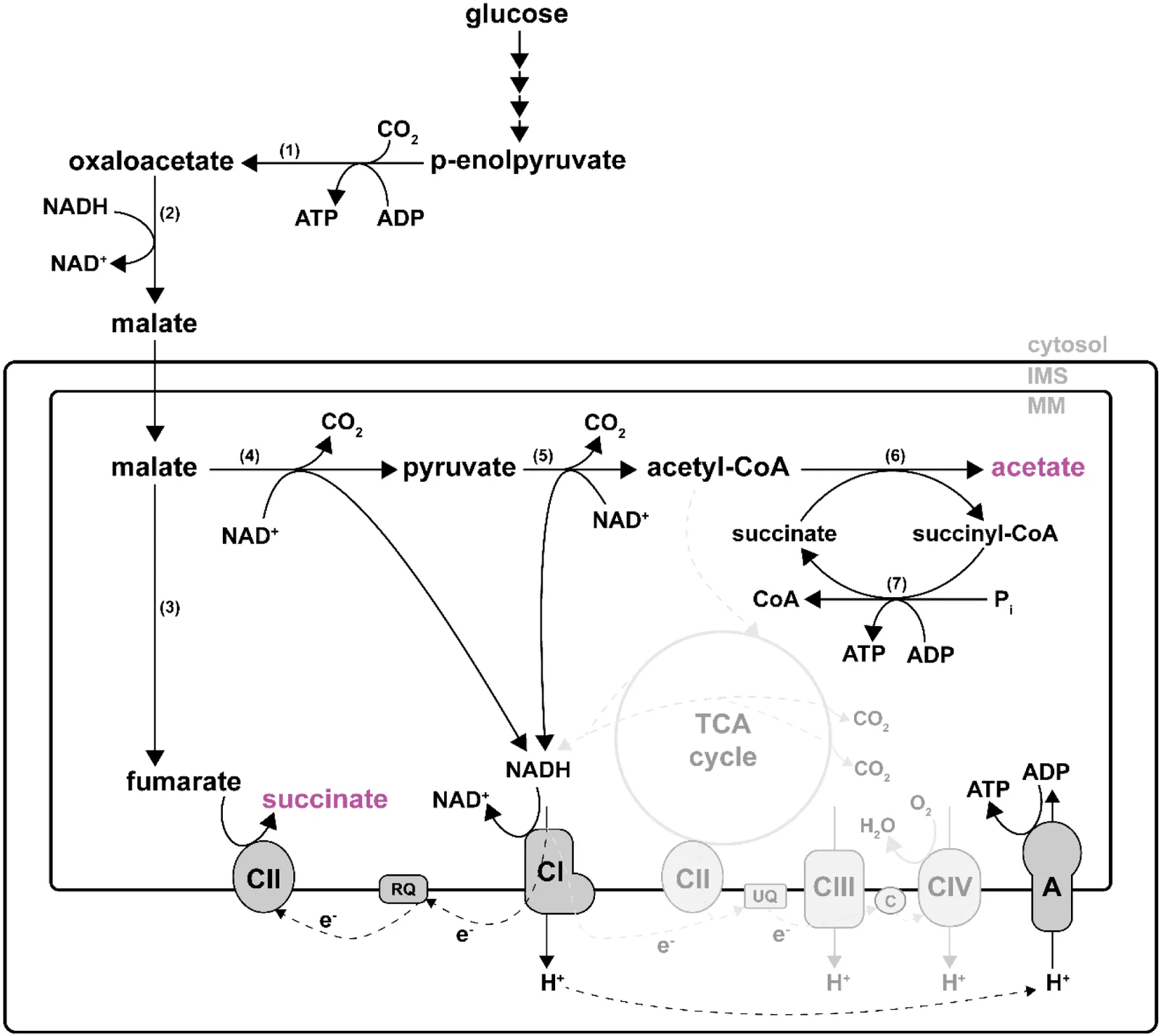
Major energy metabolism pathway, malate dismutation, in *E. multilocularis* mitochondria. Anaerobic malate dismutation is drawn in black and aerobic respiration including TCA cycle and oxidative phosphorylation shown in gray. Major end-products succinate and acetate are shown in pink-purple. A: ATPase, C: cytochrome c, CI–CIV: respiratory complexes I to IV, IMS: intermembrane space, MM: mitochondrial matrix, TCA: tricarboxylic acid cycle, RQ: rhodoquinone, UQ: ubiquinone, (1): phosphoenolpyruvate carboxykinase, (2): cytosolic malate dehydrogenase, (3): fumarase, (4): malic enzyme, (5): pyruvate dehydrogenase complex, (6): acetate:succinate CoA-transferase, (7): succinyl-CoA synthetase. Figure adapted from Ritler et al. (2019),^20^ licensed under CC BY 4.0 and figure adapted with permission from ASM 1802373–1 from Müller et al. (2012).^17^ Changes were made to the originals.

As early as 1957, it was reported that the highly related dog tapeworm *E. granulosus* secretes acetate and succinate as major end-products like many other helminths.^18^ Additionally, secretion of acetate and succinate by protoscoleces (preadult stage) of *E. granulosus* and *E. multilocularis* has been reported.^19^ More recent work showed, that also *E. multilocularis* metacestode vesicles secrete high amounts of the MD end-products acetate and succinate *in vitro*, indicative of the usage of this unconventional pathway by the parasite.^20^

MD is a relatively rare metabolic pathway, found primarily in bacteria, some unicellular eukaryotes, helminths, and certain marine invertebrates.^17^ It was thought to be absent in mammals. However, rhodoquinone, the principal electron carrier in MD, has recently been detected in mammalian mitochondria, albeit only in specific organs and at very low levels.^21^ The preferential use of rhodoquinone over ubiquinone is thought to have driven the evolution of parasite-specific enzymes in helminths, which may be exploited as pharmacological targets.^22^ Examples of this are complex I and complex II of *C. elegans*, which can be inhibited in a species-specific manner.^23,24^ Additionally, also enzymes involved in rhodoquinone synthesis such as tryptophan 2,3-dioxygenase/indoleamine 2,3-oxygenase (TDO/IDO) have been proposed as drug targets.^22^

Besides its pivotal role in the MD pathway, cMDH is also involved in other key metabolic processes including gluconeogenesis,^25^ the glyoxylate cycle^26^ and mixed acid fermentation.^27^ Structurally, MDHs belong to the lactate dehydrogenase (LDH)/ MDH superfamily which is classified into three groups: LDH (tetrameric enzymes), LDH-like MDH (tetrameric enzymes), and MDH (dimeric enzymes).^28^ Each subunit of the dimeric MDH contains two structurally and functionally distinct domains. The N-terminal domain, which contains the NAD-binding motif and the C-terminal domain that is responsible for the binding of malate and catalysis.^29^ Given its central role in parasite metabolism, inhibition of cMDH has repeatedly been proposed as a potential therapeutic strategy against helminth parasites such *Fasciola* spp.,^30^ or the protozoan parasites *Plasmodium falciparum*,^31^ *Trypanosoma brucei* or *Cryptosporidium parvum*.^32^

In this study, we report on the identification and expression of cMDH homologues in *E. multilocularis*. Furthermore, we recombinantly expressed and purified a highly expressed *E. multilocularis* cMDH (*Em*cMDH) in *Escherichia coli*, with subsequent kinetic characterization. Finally, we explored *Em*cMDH as a potential drug target, by gene silencing in the parasite as well as chemical inhibition assays on the purified enzyme and in the parasite.

## Results

### Comparative Sequence and Expression Analysis of cMDH Homologues in *E. multilocularis*

Reciprocal BLAST best hit analysis identified three cMDH homologues (EmuJ_000417100, EmuJ_000517200 and EmuJ_000893100) and two mitochondrial MDH (mMDH) homologues (EmuJ_001185000, EmuJ_001185100) in *E. multilocularis*. Phylogenetic analysis including those MDH homologues and representative metazoan orthologs (*Caenorhabditis elegans*, *Drosophila melanogaster*, *Danio rerio*, *Homo sapiens*, *Mus musculus*) resulted in two well-supported clades corresponding to cytosolic and mitochondrial enzymes, consistent with mitochondrial transfer peptide predictions (Supporting Information, Figure S1A). Focusing on the cMDH homologues, EmuJ_000417100 and EmuJ_000517200 cluster more closely with cMDH orthologs, whereas EmuJ_000893100 branches more basally, indicative of earlier evolutionary separation (Figure 2A). Furthermore, all three homologues contain the conserved N-terminal NAD­(P)-binding Rossmann-fold domain, as well as the LDH-like C-terminal domain. While EmuJ_000417100 and EmuJ_000517200 display a typical cMDH domain architecture, EmuJ_000893100 is markedly larger (494 aa) with N-terminal and C-terminal extensions (Figure 2B). Sequence identity analysis revealed that EmuJ_000417100 displays the highest similarity to canonical cMDH sequences from model organisms, with 51.9–62.1% identity to *C. elegans*, *D. melanogaster*, *D. rerio*, *H. sapiens* and *M. musculus* (Supporting Information, Figure S1B). In contrast, EmuJ_000517200 and EmuJ_000893100 showed substantially lower identities (29–34% and 11–16%, respectively) to these homologues (Supporting Information, Figure S1B), suggesting possible divergence or functional specialization. Further sequence alignment revealed that the conserved active-site residues essential for catalytic function are present in EmuJ_000417100, consistent with its high sequence identity to canonical cMDHs. In contrast, EmuJ_000517200 and EmuJ_000893100 show a lack of conservation at several key catalytic positions, suggesting that these proteins may not remain enzymatically active (Supporting Information, Figure S1C). Expression analyses using qPCR showed, that EmuJ_000417100 is the most highly expressed cMDH homologue, both in *in vitro* cultured *E. multilocularis* metacestode vesicles as well as in *in vivo* grown metacestodes (relative expression of 2.52 ± 0.53 and 1.32 ± 0.16, *in vitro* and *in vivo*, respectively, as compared to the house-keeping gene *emelp*), while the other two homologues (EmuJ_000517200 and EmuJ_000893100) show very low expression (Figure 2C). These findings indicate that EmuJ_000417100 likely represents the *bona fide* *E. multilocularis* cMDH homologue, which we termed *Em*cMDH.

**Figure 2:**
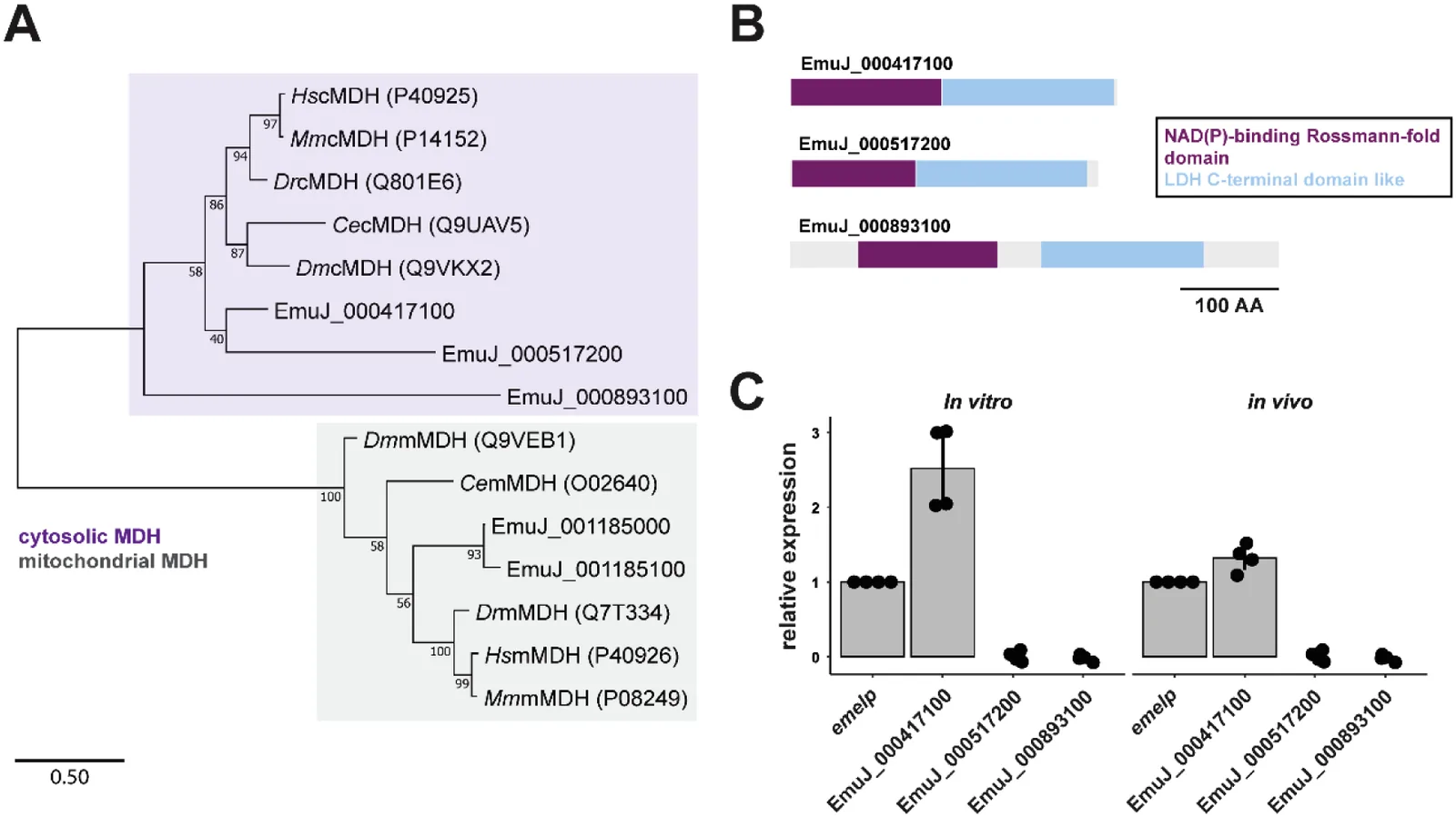
Comparative sequence and expression analysis of cMDH homologues in *E. multilocularis*. (A) Phylogenetic analysis of various cMDHs (purple) and mMDHs (gray) from model organisms *(Ce: C. elegans, Dm:* *D. melanogaster* *, Dr: D. rerio, Mm:* *M. musculus* *, Hs: H. sapiens)* as well as homologues of *E. multilocularis* (EmuJ_000417100, EmuJ_000517200, EmuJ_000893100, EmuJ_001185000, EmuJ_001185100). Numbers at the nodes represent bootstrap confidence values and the scale shows substitutions per site. Uniprot accession numbers are given in brackets. (B) Domain architecture of cMDH homologues. Shown are the identified domains, the NAD­(P)-binding Rossmann-fold domain (purple) and the LDH C-terminal like domain (light blue). Protein sequences are scaled to length. (C) Relative transcript expression of cMDH homologues of *E. multilocularis*. Expression was measured by qPCR in four biological replicates of *in vitro* cultured metacestode vesicles (*in vitro*) or metacestode tissue grown in mice (*in vivo*). Transcript *e*xpression is given relative to the house-keeping gene *emelp*. Shown are mean values ± SDs with individual data points overlaid.

### *Em*cMDH Overexpression and Kinetics

The *emcmdh* transcript was amplified from cDNA and resulted in an amplicon of the expected 999 bp (Supporting Information, Figure S2A). The amplicon was subsequently cloned into a pET151/D-TOPO vector and expressed in BL21 *E. coli*. Overexpression in BL21 *E. coli* resulted in a clear protein band at the expected 39 kDa (Supporting Information, Figure S2B). The His-tagged protein was purified using affinity chromatography, which led to a yield of around 2 mg of protein (Figure 3A).

**Figure 3:**
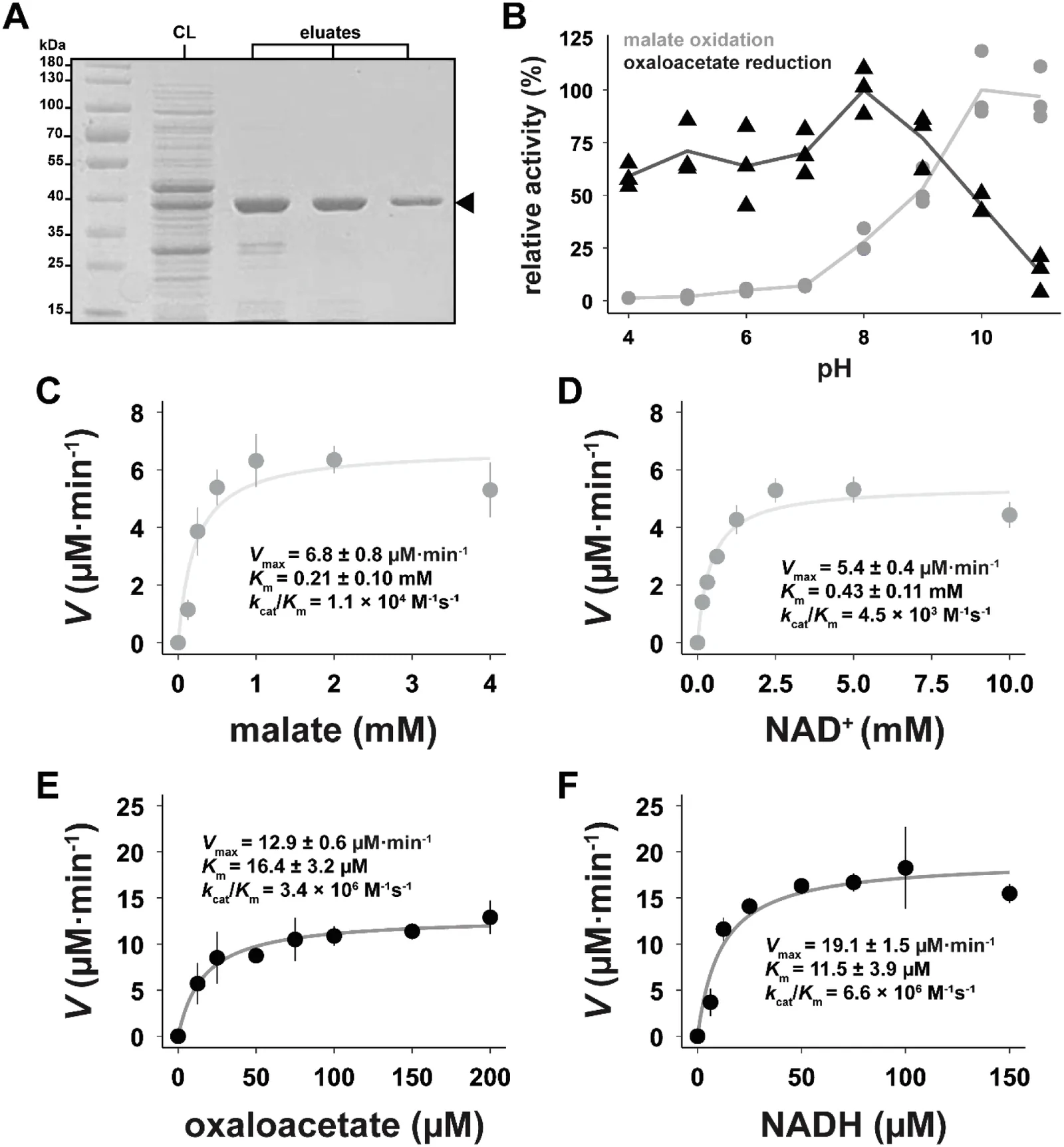
Recombinant EmcMDH kinetics. (A) Coomassie-stained SDS-PAGE gel of the affinity-purified recombinant EmcMDH. CL: clarified lysate, eluates: purified recombinant EmcMDH. Arrowhead indicates recombinant EmcMDH protein band of the expected size of 39 kDa. (B) pH-dependency of recombinant EmcMDH. Malate oxidation (gray) or oxaloacetate reduction (black) was measured under saturating substrate concentrations at various pH values. Shown are individual means of three biological replicates plotted as relatives of the respective maximum activity. (C–F) Michaelis–Menten plots malate oxidation (gray) and oxaloacetate reduction (black). Shown are means ± SD of three technical replicates with fitted Michaelis–Menten models. Kinetic parameters (*V*_max_, K_m_, *k*_cat_/K_m_) ± standard errors are listed. Titration of (C) malate, (D) NAD^+^, (E) oxaloacetate, (F) NADH.

The recombinant *Em*cMDH catalyzed both the forward (malate oxidation) and reverse (oxaloacetate reduction) reactions. The optimal pH for activity was approximately 10 in the forward reaction and 8 in the reverse reaction (Figure 3B). Catalytic efficiency (*k*_cat_/*K*_m_) was markedly lower in the forward reaction (malate oxidation), with values of 1.1 × 10^4^ M^–1^ s^–1^ for malate and 4.5 × 10^3^ M^–1^ s^–1^ for NAD^+^, compared to the reverse reaction (oxaloacetate reduction), which exhibited higher efficiencies of 3.4 × 10^6^ M^–1^ s^–1^ for oxaloacetate and 6.6 × 10^6^ M^–1^ s^–1^ for NADH. Consistently, *K*_m_ values for both substrates in the forward reaction were higher (*K*_m_ of 0.21 ± 0.10 mM and 0.43 ± 0.11 mM for malate and NAD^+^, respectively) than those determined for the reverse reaction (*K*_m_ of 16.4 ± 3.2 μM and 11.5 ± 3.9 μM for oxaloacetate and NADH, respectively), indicating a greater substrate affinity in the reduction direction (Figure 3C–F).

These findings show that recombinant *Em*cMDH is catalytically active, while oxaloacetate reduction is favored under physiological conditions *in vitro*.

### Knockdown of *Em*cMDH Leads to Impaired Metacestode Vesicle Development

To investigate the importance of *Em*cMDH for parasite development, RNAi was performed on GL cells with siRNA targeting the *emcmdh* transcript (*cmdh*) or a nontargeting control siRNA against the green fluorescent protein (*gfp*) of the cnidarian *Aequorea victoria* of which no ortholog is present in *E. multilocularis*. The RNAi-mediated knockdown of *emcmdh* was confirmed by qPCR, which showed that relative *emcmdh* transcript expression was reduced to 0.18 ± 0.02 compared to the nontargeting *gfp* control 3 days after electroporation (Figure 4A). Knockdown also led to a stable and long-lasting reduction of relative *Em*cMDH protein levels of 0.06 compared to the control 24 days after electroporation (Figure 4B). These results confirm the effectiveness of the knockdown approach and validate the specificity of the targeting strategy. Importantly, knockdown of *emcmdh* did not lead to upregulation of the other two identified cMDH paralogs EmuJ_000517200 and EmuJ_000893100 (Figure 4C). Despite successful knockdown, GL cells were still able to form cell aggregates and formed metacestode vesicles upon prolonged cultivation. However, the relative metacestode vesicle formation rate was lower (0.66 ± 0.02) compared to the *gfp* control, highlighting the importance of *Em*cMDH for parasite development (Figure 4D).

**Figure 4:**
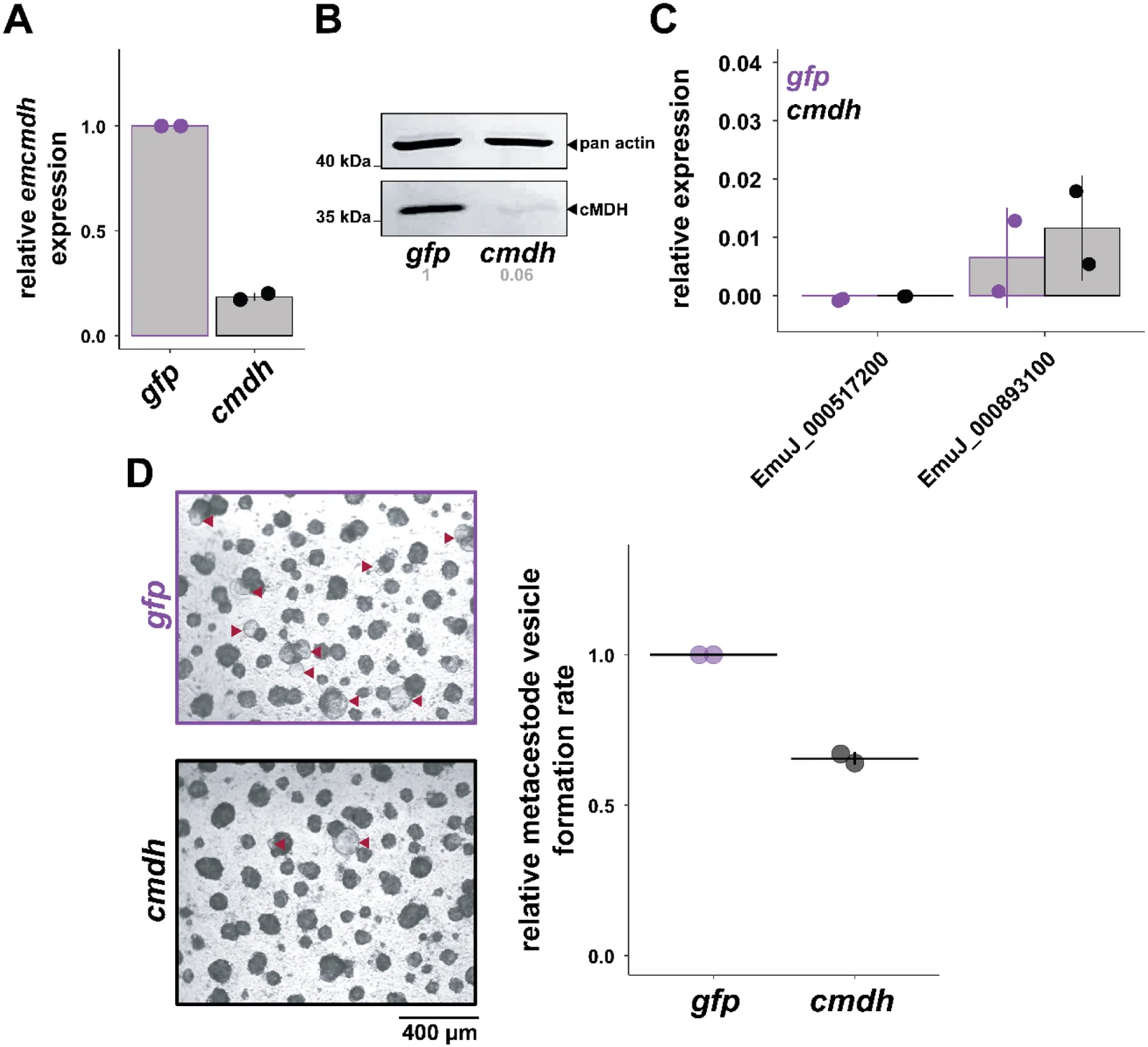
Knockdown of EmcMDH in *E. multilocularis* GL cells. GL cells were treated with 2 μM siRNA targeting emcmdh (cmdh; black) or the nontargeting siRNA against green fluorescent protein (gfp; purple) of the cnidarian Aequorea victoria of which no ortholog is present in *E. multilocularis*. (A) Relative expression of the emcmdh transcript 3 days after electroporation. Shown are means ± SD of two independent experiments with individual data points overlaid. Emcmdh expression is given relative to the control gfp. (B) Representative Western blot of EmcMDH 24 days after electroporation. Relative quantification of EmcMDH is given in gray numbers. (C) Relative expression levels of emcmdh paralogs 3 days after electroporation. Shown are means ± SD of two independent experiments with individual data points overlaid. Transcript expression is given relative to the house-keeping gene emelp. (D) Left, representative pictures of GL cell populations 18 days after electroporation. Burgundy arrowheads indicate emerging metacestode vesicles. Right, relative metacestode vesicle formation rate. Shown are means ± SD of two independent experiments, each performed with technical quadruplicates.

### Gossypol Is a Potent *Em*cMDH Inhibitor and Is Active against *E. multilocularis*

Gossypol, a natural polyphenolic compound, derived from cotton plants (*Gossypium* spp.) was previously described to be an oxidoreductase inhibitor.^33^ We found that gossypol potently inhibited the recombinant *Em*cMDH with an IC_50_ of 0.25 ± 0.04 μM. In contrast, recombinant cMDH from *M. musculus* (*Mm*cMDH) (Supporting Information, Figure S2E) was inhibited approximately 80-fold less potently (IC_50_ of 20.13 ± 2.46 μM), indicating a parasite-specific activity (Figure 5A). To validate gossypol’s action on native *Em*cMDH, it was tested in cytosolic fractions of *E. multilocularis* metacestode vesicles. Purity of cytosolic fractions was validated using Western blot analysis (Supporting Information, Figure S3). Gossypol significantly inhibited *Em*cMDH activity in cytosolic fractions and reduced relative activity to 56.7 ± 9.7% compared to the solvent control DMSO (*p* = 0.019, 95% CI: 12.20 – 74.42) (Figure 5B). Employing a high-confidence AlphaFold-predicted structure of *Em*cMDH (Supporting Information, Figure S4), molecular docking analyses predicted that gossypol binds to the NAD-binding site of *Em*cMDH, positioning gossypol within 5 Å of key NAD-associated residues (G11, A13, G14, I16, D42, V87, G88, A89, and H187) and hydrogen bond formation between gossypol and residues Q15 and S241, which participate in NAD binding, as well as with residue S242, which is involved in malate binding (Figure 5C).

**Figure 5:**
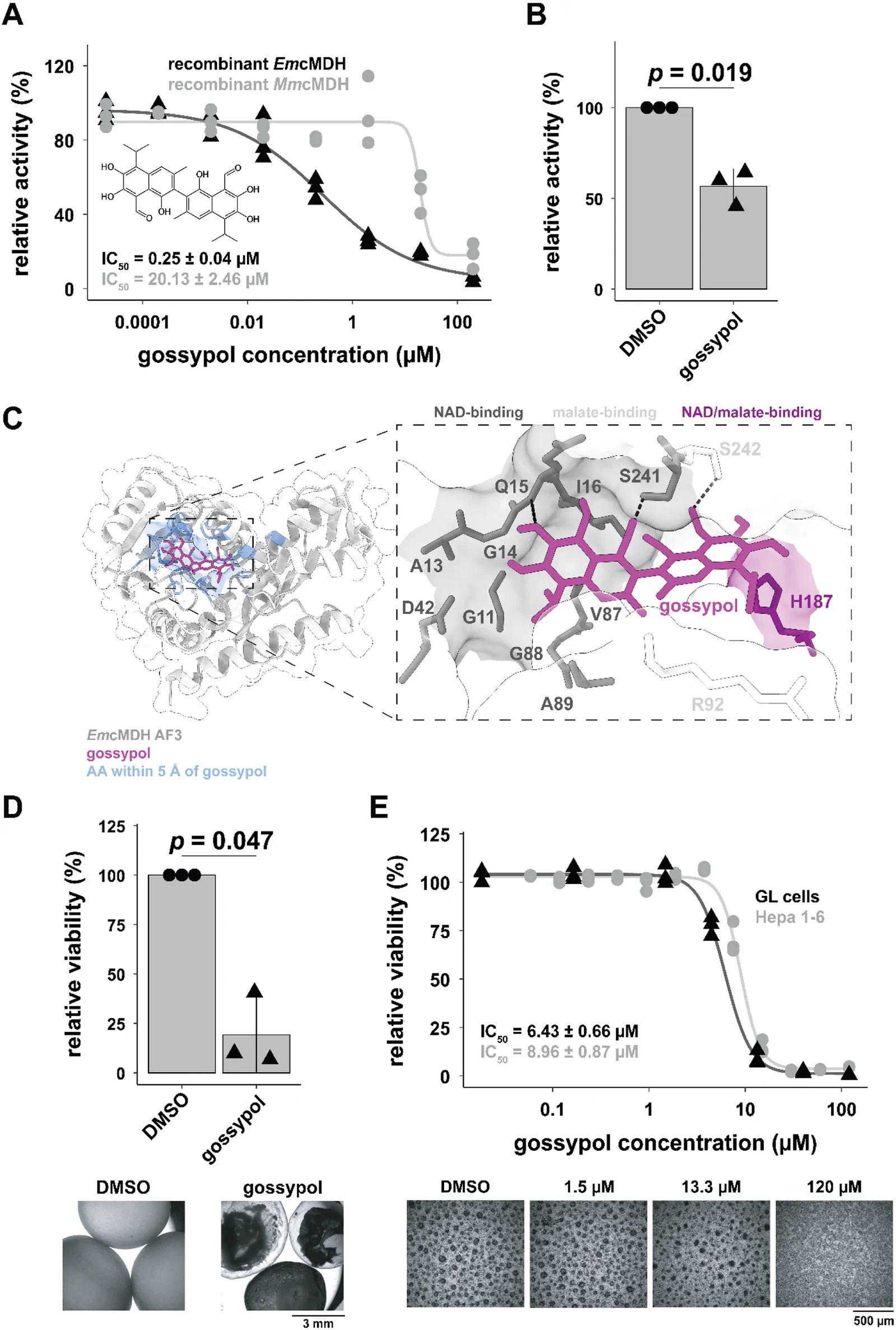
Gossypol inhibits EmcMDH and is active against *E. multilocularis* metacestode vesicles and GL cells. (A) Efficacy of gossypol against recombinant cMDHs (EmcMDH; black and MmcMDH; gray). Shown are individual means of three biological replicates fitted to a four-parameter log–logistic model. The reported IC_50_ values represent mean ± SD of three independent biological replicates. Additionally shown is the chemical structure of gossypol. (B) EmcMDH inhibition in cytosolic fractions of *E. multilocularis* metacestode vesicles. EmcMDH activity was measured in cytosolic fractions treated with 20 μM gossypol or the solvent control DMSO. Shown are three independent experiments. Differences were assessed using Welch’s *t* test and results were considered statistically significant at *p* < 0.05. (C) Predicted EmcMDH-gossypol complex. Shown is gossypol (magenta), the gossypol-binding pocket (AA residues within 5 Å of gossypol; blue). Close-up shows gossypol (magenta), NAD-binding residues (dark gray), malate-binding residues (light gray) and residues involved in binding of both substrates (purple), based on data previously published.^34^ Hydrogen bonds are represented as dashed black lines. (D) Top, relative viability of metacestode vesicles treated with either DMSO or 20 μM gossypol. Shown are three independent experiments. Differences were assessed using Welch’s *t* test and results were considered statistically significant at *p* < 0.05. Bottom, representative micrographs of metacestode vesicles treated with either DMSO or 20 μM gossypol. (E) Top, efficacy of gossypol against GL cells (black) and mammalian cells (Hepa 1-6; gray). Shown are individual means of three biological replicates fitted to a four-parameter log–logistic model. The reported IC_50_ represents the mean ± SD of the three independent biological replicates. Bottom, shown are representative micrographs of GL cell aggregates treated with gossypol as depicted.

In cellular assays, gossypol showed efficacy against metacestode vesicles. Morphologically, it led to the detachment of the GL from the laminated layer, indicative of parasite death and it significantly reduced relative viability to 19.19 ± 18.71% compared to the DMSO control (*p* = 0.047, 95% CI: −5.25 to 154.86), when tested at 20 μM (Figure 5D). Furthermore, gossypol exhibited parasiticidal activity against *E. multilocularis* GL cells with an IC_50_ of 6.43 ± 0.66 μM, demonstrating cellular activity consistent with cMDH inhibition. At concentrations from 13.3 μM, GL cells displayed pronounced morphological changes, including loss of GL cell aggregate integrity with a complete abolition of aggregate formation at 120 μM (Figure 5E). Against mammalian Hepa 1-6 cells, gossypol showed modest toxicity (IC_50_ of 8.96 ± 0.87 μM), resulting in a ∼1.4-fold selectivity index as compared to *E. multilocularis* cells (Figure 5E). While selectivity remained limited, these data establish gossypol as an active *Em*cMDH inhibitor with proof-of-concept parasite activity.

## Discussion

AE is a severe zoonotic disease with low but increasing case numbers. Nowadays, drug treatment options are limited and not curative, thus in nonoperable cases AE is a chronic, life-altering disease. Increasing the knowledge of the fundamental biochemistry and metabolism of this parasite may open doors for new drug treatment avenues, such as the unconventional MD pathway, which plays a major role in anaerobic energy metabolism and is largely absent in the mammalian host. cMDH is thought to play a central role in MD and based on the data presented in this study we propose cMDH as a potential metabolic drug target in AE.

Bioinformatic analyses led to the identification of three cMDH homologues (EmuJ_000417100, EmuJ_000517200 and EmuJ_000893100), which clearly clustered with metazoan cMDH orthologs. All three homologues displayed characteristic MDH domain architecture, however, EmuJ_000517200 and EmuJ_000893100 showed sequence divergence, potentially reflecting specialized functions, warranting further investigation. Importantly, our expression data establishes EmuJ_000417100 (termed *Em*cMDH) as the primary cMDH in *E. multilocularis*, with high expression across both *in vitro* metacestode vesicle cultures and *in vivo* parasite lesions, while EmuJ_000517200 and EmuJ_000893100 exhibited low expression levels. The consistent dominance observed by qPCR validates patterns in independent RNA-seq data sets,^35^ in which the *emcmdh* transcript ranks among the most highly expressed genes in metacestode vesicles (56th of 10,669 detected transcripts, in *in vitro* cultured metacestode vesicles). Furthermore, high expression of *emcmdh* and low expression of EmuJ_000517200 and EmuJ_000893100 is observed in various developmental stages of the parasite including protoscoleces and adult worms, besides metacestode vesicles, underscoring its central metabolic importance throughout parasite development.^35^ However, transcript abundance alone does not exclude potential stage-specific, condition-dependent, or specialized functions of the lowly expressed paralogs. Furthermore, previously published proteomic data corroborates the dominance of *Em*cMDH, which ranked among the most highly expressed proteins (12th of 2,954 detected, in *in vitro* cultured metacestode vesicles), while EmuJ_000517200 and EmuJ_000893100 were not detectable.^36^ This positions *Em*cMDH as a central metabolic enzyme during the clinically relevant metacestode stage.

Due to the high expression of *Em*cMDH in metacestode vesicles, we further focused on this protein and it was recombinantly expressed in *E. coli*. Biochemical characterization of the recombinant *Em*cMDH validated its enzymatic activity. Interestingly, malate oxidation activity was maximal at pH 10, while maximal oxaloacetate reduction was observed at a physiological pH of 8. The optimal malate oxidation pH is well above the physiological intracellular pH of the parasite. Therefore, the alkaline optimum is unlikely to represent the actual operating conditions *in vivo*. Instead, it probably reflects the intrinsic catalytic properties of the enzyme, including the protonation state of active-site residues and the reaction mechanism. Recombinant *Em*cMDH exhibits classical cMDH kinetics, where oxaloacetate reduction is favored. This is in accordance with previously characterized cMDHs, such as the helminth cMDHs of *Fasciola gigantica*,^30^ *Spirometra mansoni*,^37^ *Clonorchis sinensis* ^38^ *or* *Taenia solium* ^34^ but also mammalian cMDHs such as porcine cMDH^39^ or human cMDH^40^ (kinetic parameters are listed in Supporting Information, Table S1). Successful purification of recombinant *Em*cMDH and the establishment of enzymatic assays lay the foundation for future target-based drug screening for potent and specific *Em*cMDH inhibitors.

Importance of *Em*cMDH was demonstrated genetically using RNAi-mediated knockdown in GL cells. Knockdown was successful both on a transcript level as well as on a protein level, where it led to a stable and long-lasting reduction of *Em*cMDH. Importantly, the absence of significant upregulation of the other predicted cMDH paralogs, upon *Em*cMDH knockdown suggests that these paralogs do not compensate for the loss of *Em*cMDH. This lack of redundancy highlights a potential metabolic vulnerability, indicating that the parasite relies predominantly on a single cytosolic isoform to sustain its metabolic needs under the tested conditions. Functionally, knockdown of *Em*cMDH reduced metacestode vesicle formation in GL cells, providing evidence that *Em*cMDH contributes to parasite growth and development. Although vesicle formation was markedly impaired, it was not completely abolished, indicating that *Em*cMDH is important for efficient metacestode development but may not be strictly essential under the experimental conditions applied. The residual capacity for vesicle formation may reflect incomplete knockdown efficiency or the ability of the parasite to partially compensate through alternative metabolic pathways or redundant enzymatic activities. Nevertheless, the observed reduction in metacestode vesicle number suggests impaired cellular proliferation and/or differentiation, processes that are likely dependent on an efficient energy metabolism. Given the central role of MD in helminth anaerobic respiration, disruption of cMDH likely compromises redox balance and energy production, ultimately limiting the developmental capacity of the parasite. The prolonged culture periods required for metacestode vesicles, together with the limited availability of parasite material, restrict the feasibility of experimental replication and downstream analyses following RNAi knockdown, thereby limiting the scope of the current study. Thus, future experiments should be undertaken to measure residual *Em*cMDH activity as well as metabolomic analyses, including the quantification of pathway intermediates (e.g., malate, oxaloacetate, succinate, and acetate) and assessment of cellular redox balance following *Em*cMDH knockdown or chemical inhibition. These experiments would provide valuable mechanistic insight into the metabolic consequences of *Em*cMDH disruption and would help determine whether cMDH represents a rate-limiting step in the MD pathway and an effective target for therapeutic intervention.

*Em*cMDH was explored as a potential drug target and chemical inhibition of *Em*cMDH was demonstrated by the oxidoreductase inhibitor gossypol.^33^ Interestingly, gossypol, which was predicted to bind to the conserved NAD-binding pocket, exhibited remarkable parasite-specificity. In our hands, murine cMDH was inhibited with an IC_50_ of around 20 μM, whereas on human spermatozoal MDH was reported to display IC_50_ values around 1.2–2.9 μM.^41^ While part of this discrepancy may reflect differences in assay format, it may also result from subtle sequence and structural divergence within and around the Rossmann-fold NAD-binding region of mammalian enzymes, which can markedly influence gossypol affinity. Furthermore, gossypol inhibited *Em*cMDH in cytosolic fractions, demonstrating on-target activity in the parasite. Thus, gossypol may act as a starting point for chemical optimization and hints at exploitable differences between parasite and mammalian cMDH. As a proof-of-concept, gossypol was evaluated against *E. multilocularis* metacestode vesicles as well as GL cells, where it demonstrated potent activity, in accordance with anticestodal effects of gossypol described against *Taenia taeniaeformis* metacestodes *in vitro* ^42^ as well as protective effects in a rodent model.^43^ However, gossypol lacked parasite-specificity on a cellular level as it also exhibited mammalian cell toxicity. This lack in specificity may be explained by the promiscuity of gossypol. Like many compounds derived from plants, gossypol has many molecular targets. Early studies reported its inhibitory effects on several oxidoreductases, such as LDH, MDH and glutamate dehydrogenase isoforms in humans,^44^ as well as the related enzymes α-hydroxy-acid dehydrogenase, MDH and glutamate dehydrogenase in the unicellular parasite *Trypanosoma cruzi* ^45^ or inhibition of LDH and cytosolic MDH in *Plasmodium falciparum*.^46^ As gossypol is a general oxidoreductase inhibitor, other oxidoreductases, such as the thioredoxin glutathione reductase, succinate dehydrogenase complex or threonine dehydrogenase, all of which were described to be potential drug targets in *Echinococcus* spp.,^47–49^ may be targeted by gossypol. Further research has identified additional targets, including lipoxygenases,^50^ DNA polymerase α^51^ or the antiapoptotic protein Bcl-xL.^52^ The latter is particularly relevant to the well-documented anticancer activity of gossypol, which has led to its evaluation in multiple clinical trials.^53^ Beyond its anticancer activity, gossypol was first shown to exert antifertility effects, largely attributed to inhibition of LDH isoforms in spermatozoa.^54^ Furthermore, gossypol has been shown to exhibit antiviral,^55,56^ antiparasitic^45,57,58^ and antimicrobial effects.^59,60^ Thus, although gossypol provides proof-of-concept that chemical inhibition of *Em*cMDH can impair parasite viability, its well-documented promiscuity precludes attributing its antiparasitic activity solely to this target. Future work should therefore focus on the identification and optimization of more selective *Em*cMDH inhibitors through structure-based drug design or high-throughput screening approaches. Future structural comparison of the parasite and mammalian cMDH binding sites would further help to identify exploitable differences that could guide the development of parasite-selective inhibitors.

## Conclusions

This work established *Em*cMDH as a central and druggable enzyme in the unusual MD pathway of the cestode parasite *E. multilocularis*. Expression analysis, biochemical characterization, RNAi and inhibitor assays demonstrated that *Em*cMDH is both highly expressed, active under physiologically relevant conditions and vulnerable to small-molecule inhibition, which led to marked defects in parasite viability. In conclusion, our data identify *Em*cMDH as a potential metabolic drug target in AE, but further validation in animal infection models and the identification of selective inhibitors remain necessary, providing a framework for the future development and optimization of MDH-directed anthelmintic compounds.

## Materials and Methods

### Chemicals and Reagents

Unless otherwise specified, all chemicals were obtained from Sigma-Aldrich (Buchs, Switzerland), and all plastic ware was sourced from Sarstedt (Sevelen, Switzerland). Dulbecco’s Modified Eagle Medium (DMEM) as well as penicillin-streptomycin (10,000 Units/mL penicillin and 10,000 μg/mL streptomycin) were acquired from Gibco (Fisher Scientific AG, Reinach, Switzerland). Fetal bovine serum (FBS) and Trypsin/EDTA solution (0.05% Trypsin and 0.02% EDTA) were purchased from Bioswisstec (Schaffhausen, Switzerland). Reuber rat hepatoma (RH) cells (H-4-II-E) were obtained from ATCC (Molsheim Cedex, France). Gossypol ((±)-gossypol, BL 193) was purchased from MedChemExpress (Sollentuna, Sweden).

### Mice and Ethics Statement

*E. multilocularis* strain H95 was propagated in female BALB/c mice (Charles River Laboratories, Sulzheim, Germany) following the protocol previously described.^49^ Animals were housed under standardized conditions, including a 12-h light/dark cycle, ambient temperatures of 21–23 °C, and relative humidity levels of 45–55%. Food and water were provided *ad libitum*. Cage enrichment included nestlets (Plexx, Elst, Netherlands), mouse houses (Tecniplast, Gams, Switzerland), and tunnels (Zoonlab, Castrop-Rauxel, Germany). All procedures were approved by the Animal Welfare Committee of the canton of Bern under license numbers BE47/25 and BE2/22, and carried out in accordance with the Swiss Federal Protection of Animals Act (TSchV, SR455).

### *In Vitro* Culture of *E. multilocularis* metacestode Vesicles

*E. multilocularis* metacestode vesicles were cultured following the described method.^49^ In brief, parasite tissue was collected from the peritoneal cavity of infected BALB/c mice and passed through a tea strainer (Migros, Bern, Switzerland). The material was then incubated overnight at 4 °C in PBS supplemented with penicillin (100 U/mL), streptomycin (100 μg/mL), and tetracycline (10 μg/mL). On the following day, 1.5 mL of the parasite preparation was cocultured with RH cells in DMEM containing 10% FBS, penicillin (100 U/mL), streptomycin (100 μg/mL), and tetracycline (5 μg/mL) at 37 °C in a humidified incubator with 5% CO_2_.

### Identification of Malate Dehydrogenases in *E. multilocularis*

Protein sequences (obtained from UniProt) of cytosolic as well as mitochondrial MDHs of reference organisms (*Caenorhabditis elegans*, *Drosophila melanogaster*, *Danio rerio*, *Homo sapiens* *and* *Mus musculus*) were blasted against the protein database of *E. multilocularis* PRJEB122 ^61^ using BioEdit (Version 7.2). Top hits within the protein database of *E. multilocularis* were then blasted reciprocally against the NCBI nonredundant protein database of the reference organisms (https://blast.ncbi.nlm.nih.gov/Blast.cgi). Mitochondrial transfer peptide probability was predicted using TargetP 2.0.^62^ Domain architecture of putative cMDHs was done using InterPro.^63^ Phylogenetic analysis was performed using MEGA11 software^64^ using the maximum likelihood algorithm, 1000 bootstrap replicates, and the JTT substitution model.^65^ Multiple sequence alignment of cMDH homologues of *E. multilocularis* and reference organisms was generated using BioEdit applying the default ClustalW algorithm^66^ and visualized using ESPript 3.0 ^67^ with secondary structure information retrieved from *Hs*cMDH (PDB: 7RM9).^40^

### Expression of Malate Dehydrogenases in *E. multilocularis*

#### Preparation of *E. multilocularis* Metacestode Vesicles and *In Vivo* Grown Metacestodes

*E. multilocularis* metacestode vesicles of approximately six months of age and a diameter of 5 mm were purified and transferred to an axenic culture system devoid of RH cells, following the protocol previously described.^68^ The metacestode vesicles were mixed with three volumes of conditioned medium, and 4 mL of this suspension were distributed into 6-well plates per well and incubated for 5 days under a humid, microaerobic atmosphere (85% N_2_, 10% CO_2_, and 5% O_2_). After incubation, metacestode vesicles were washed with PBS, mechanically disrupted, and washed again with PBS. The resulting vesicle tissue was resuspended in TRI Reagent, agitated at 1,400 rpm for 15 min at room temperature, and stored at −20 °C until RNA isolation. For parasites grown intraperitoneally, metacestode tissue from four individual mice was washed with PBS, mechanically disrupted, and suspended in TRI Reagent. Homogenization was performed by adding a 5 mm glass bead to each tube and five cycles at 4 m/s for 20 s each using a FastPrep-24 Classic instrument (MP Biomedicals, Illkirch-Graffenstaden, France). Following this, samples were agitated at 1,400 rpm for 15 min at room temperature and then centrifuged at 12,000 × g for 10 min at 4 °C. The resulting supernatant was transferred to a fresh tube and stored at −20 °C until RNA isolation.

#### RNA Isolation and cDNA Synthesis

Total RNA of axenically cultured *E. multilocularis* metacestode vesicles and *ex vivo* metacestode tissue was extracted using a phenol/chloroform-based method as described.^49^ After RNA isolation, 1 μg of RNA was reverse transcribed into cDNA using the GoScript Reverse Transcription System (Promega, Dübendorf, Switzerland) in a total reaction volume of 20 μL. RNA isolation and cDNA synthesis were performed from four independent biological replicates of *in vitro* cultured metacestode vesicles and from parasite material obtained from four individual mice for the *in vivo* samples.

#### RT-qPCR

Gene expression analysis was performed using specific intron-flanking primers listed in Supporting Table 2. Quantitative real-time PCR was conducted using the QuantiTect SYBR Green PCR Kit (Qiagen, Steinhausen, Switzerland) on a CFX Opus 96 Real-Time PCR System (Bio-Rad) in a final volume of 10 μL with the following settings: initial activation of the polymerase at 95 °C for 15 min, followed by 40 cycles of denaturation for 15 s at 95 °C, annealing at 62.5 °C for 30 s, elongation for 30 s at 72 °C. In the end, a melting curve analysis (65 to 95 °C, 0.5 °C increase per 5 s) of the amplified product was performed to confirm the specificity of the primers for their target. Primer efficiencies were determined by running RT-qPCR reactions with titrations of cDNA (Supporting Table 2). Expression was analyzed in technical triplicates of four independent biological replicates and calculated relative to the house-keeping gene *emelp* ^49^ while correcting for the respective primer efficiency.

### Molecular Cloning, Overexpression, and Purification of *Em*cMDH and *Mm*cMDH

The full coding sequence of *emcmdh* (EmuJ_000417100) was amplified from cDNA generated from *in vitro* grown *E. multilocularis* metacestode vesicles. *Mmcmdh* (ENSMUST00000239073.2) was amplified from cDNA generated from cultured murine Hepa 1-6 cells (Hepa 1-6 cells were cultured in DMEM containing 10% FBS, penicillin (100 U/mL), streptomycin (100 μg/mL), and tetracycline (5 μg/mL) at 37 °C in a humidified incubator with 5% CO_2_. Total RNA was isolated using the Direct-zol RNA miniprep kit (Zymo Research, Lucerna-Chem AG, Lucerne, Switzerland) and cDNA was synthesized using the GoScript Reverse Transcription System (Promega, Dübendorf, Switzerland). The 999 and 1,005 bp long amplicons were subsequently cloned into the pET151/D-TOPO vector using the Champion pET151 Directional TOPO Expression Kit (Fisher Scientific AG, Reinach, Switzerland). The recombined plasmids were used to transform *E. coli* TOP10 (Fisher Scientific AG, Reinach, Switzerland) according to the manufacturer’s protocol. Positive clones were selected via colony PCR using a vector-specific as well as an insert-specific primer. Plasmid DNA was extracted using the ZymoPURE Plasmid Miniprep Kit (Zymo Research, Lucerna-Chem AG, Lucerne, Switzerland) and Sanger sequencing was conducted at Microsynth AG (Balgach, Switzerland). Correct plasmids were used to transform *E. coli* BL21 (Fisher Scientific AG, Reinach, Switzerland) for protein overexpression and the recombinant his-tagged proteins were purified using the Macherey-Nagel Protino Ni-TED-IDA 1000 Kit (Fisher Scientific AG, Reinach, Switzerland). Purity of eluates was assessed using SDS-PAGE and protein concentration was measured via BCA assay using the Pierce BCA Protein Assay Kit (Fisher Scientific AG, Reinach, Switzerland).

### Enzymatic Assay of *Em*cMDH

#### *Em*cMDH pH Dependency

To test *Em*cMDH pH dependency, enzymatic activity was measured in technical triplicates at different pH values (4–11) in 50 mM citrate-glycine-HEPES buffer including 150 mM NaCl using 5 nM purified *Em*cMDH and 150 μM of both substrates for oxaloacetate reduction and 50 nM purified *Em*cMDH and 5 mM of both substrates for malate oxidation, respectively. Enzymatic activity was measured via the fluorescence of NADH at 460 nm at 25 °C on a HIDEX Sense microplate reader (Hidex, Turku, Finland). Activity was calculated relative to the maximal activity observed at the respective pH values. This experiment was performed three times independently.

#### *Em*cMDH Kinetics

Enzyme kinetics were performed in 50 mM Tris-HCl pH 8.0 buffer at 25 °C in technical triplicates using 50 nM of purified *Em*cMDH for malate oxidation and 5 nM of purified *Em*cMDH for oxaloacetate reduction. To determine the kinetics of malate oxidation, both substrates, NAD^+^ and malate were titrated from 10 to 0.15 mM and 4 to 0.12 mM, respectively, while having a constant concentration of 5 mM of the respective cosubstrate. Similarly, to determine the kinetics of oxaloacetate reduction, both substrates NADH (Carl Roth AG, Arlesheim, Switzerland) and oxaloacetate (Carl Roth AG) were titrated from 150 to 6.2 μM and 200 to 12.5 μM, respectively, while having a constant concentration of 150 μM of either cosubstrate. Reaction rate (*V*) was calculated by interpolation to a NAD^+^/NADH standard curve.

### RNAi-Mediated Knockdown of *Em*cMDH

#### Isolation of GL Cells from Metacestode Vesicles

GL cells were isolated as described.^69^ Based on BCA assay measurements using the Pierce BCA Protein Assay Kit, one AU was equivalent to 1.11 ± 0.19 μg of total protein. For cultivation, 1,000 AU of GL cells were cultured overnight in 5 mL of cDMEM at 37 °C under a humid nitrogen atmosphere. The following day, 2,000 AU of GL cells were combined and cultured for an additional 3 h at 37 °C in the same atmosphere.

### Electroporation and Subsequent Cultivation of GL Cells

RNAi-mediated knockdown was performed as described.^70^ A 23 bp siRNA targeting *emcmdh* (EmuJ_000417100) was designed using siDirect 2.1 ^71^ and ordered as a desalted duplex siRNA (Merck, Darmstadt, Germany). Additionally, a siRNA targeting green fluorescent protein *(gfp)* of the cnidarian *Aequorea victoria*, of which no ortholog is present in *E. multilocularis*, was applied as a control (sequences are shown in Supporting Table 2).^70^ The GL cell suspension was harvested and spun down at 200 × g for 2 min at room temperature and the cell pellet was washed with electroporation buffer. After centrifuging again, the cell pellet was resuspended in electroporation buffer containing 2 μM of the respective siRNA. The cell suspension was placed into a 1 mm gap electroporation cuvette (Fisher Scientific AG, Reinach, Switzerland) and pulsed once at 200 V, 13 Ω and 50 μF using aBTX electro cell manipulator 600. After incubation of 15 min at 37 °C the cell suspension was centrifuged at 200 × g for 2 min and the cell pellet was resuspended in cDMEM and distributed into a 96-well plate with 150 AU per well and cultivated under a low oxygen atmosphere (85% N_2_, 10% CO_2_, and 0–5% O_2_). For prolonged cultivation of these GL cells, half of the medium was replaced three times a week. GL cells were cultured for two and a half to 3 weeks and after two and a half to 3 weeks, newly formed metacestode vesicles were counted in a blinded manner. The experiment was conducted in technical quadruplicates and performed twice independently. The number of independent experiments was limited by the 6–12 month culture period required for the model and the limited availability of parasite material.

### RNA Isolation of GL Cells and cDNA Synthesis

Samples for RNA isolation were taken 3 days after electroporation. 600 AU of GL cells were combined and spun down at 600 × g for 2 min. The pellet was washed 2 times with ice-cold PBS and finally resuspended in 500 μL TRI Reagent. Total RNA was isolated using the Direct-zol RNA Miniprep Kit (Zymo Research, Lucerna-Chem AG, Lucerne, Switzerland) according to the manufacturer’s protocol. 0.5 μg of RNA was reverse transcribed into cDNA using the GoScript Reverse Transcription System (Promega, Dübendorf, Switzerland) in a total reaction volume of 20 μL.

### RT-qPCR

Gene expression analysis was performed as described for metacestode vesicles above.

### Western Blotting

Generation of a whole cell lysate was done as previously described with some alterations.^72^ After 3 weeks of cultivation, 600 AU were combined and spun down at 600 × g for 2 min. The pellet was washed 2 times with ice-cold PBS and finally the pellet was resuspended in 150 μL RIPA buffer (50 mM Tris-HCl pH 7.4, 1 mM EDTA, 150 mM NaCl, 0.1% SDS, 0.5% DOC, 1% Tx-100) supplemented with Halt Protease Inhibitor Cocktail (Fisher Scientific AG, Reinach, Switzerland). The suspension was then incubated on ice for 40 min with regular vortexing and spun down at 16,000 × g for 15 min at 4 °C. Protein concentration of the supernatant was measured using Pierce BCA Protein Assay kit (Fisher Scientific AG, Reinach, Switzerland) according to the manufacturer’s protocol. Whole cell lysates (7.5 μg per lane) were separated on a 10% SDS-PAGE gel and transferred onto a nitrocellulose membrane. The membrane was blocked for 1 h at RT in 2% dry milk 0.1% PBS-Tween 20. After blocking, the membrane was probed using an anti-MDH1 polyclonal antibody (E-AB-62826, Elabscience, Houston, USA) or an anti-pan actin monoclonal antibody (Ab-5, Fisher Scientific AG, Reinach, Switzerland) diluted 1:1,000 in 2% dry milk 0.1% PBS-Tween 20 at 4 °C overnight. Before addition of the secondary antibodies, the membrane was extensively washed using 0.1% PBS-Tween 20. Secondary antibodies, anti-rabbit IgG AP conjugate (Promega, Dübendorf, Switzerland) or anti-mouse IgG AP conjugate (Promega, Dübendorf, Switzerland), diluted 1:2,000 in 2% dry milk 0.1% PBS-Tween 20 were added to the membrane for 1h at RT and subsequently, the membrane was washed extensively with PBS. For detection, membranes were incubated in AP buffer (100 mM Tris-HCl pH 9.5, 100 mM NaCl, 5 mM MgCl_2_) supplemented with 3 μL/mL NBT/BCIP stock solution (Roche, Basel, Switzerland). Protein band intensities were quantified using Fiji (ImageJ 1.54p). Protein expression levels were normalized to the loading control (pan actin) and given as a relative of the control *gfp*.

### Inhibitor Assays

#### *Em*cMDH and *Mm*cMDH Inhibition Assays

Gossypol, a potent oxidoreductase inhibitor,^33^ was tested against the recombinant cMDHs (both *Em*cMDH and *Mm*cMDH). Gossypol was tested in a 1:10 serial dilution from 200 to 2 × 10^–6^ μM. Oxaloacetate reduction was monitored via the fluorescence of NADH at 460 nm at 25 °C in 50 mM Tris-HCl pH 8.0 with 150 μM of both cosubstrates and 5 nM recombinant cMDH. Enzyme activity was calculated as a relative of the solvent control DMSO. IC_50_ values were determined by fitting dose–response curves from three independent experiments, each performed with three technical replicates, to a four-parameter log–logistic model separately. The reported IC_50_ values represent the mean ± SD of the three independent replicates.

#### *Em*cMDH Inhibition in Cytosolic Fractions of *E. multilocularis* metacestode Vesicles

The on-target activity of gossypol was tested in cytosolic fractions of *E. multilocularis* metacestode vesicles. Cytosolic fractions were generated as described previously^73^ with some adaptations. *E. multilocularis* metacestode vesicles were transferred to an axenic culture system.^68^ Approximately one mL of metacestode vesicles were ruptured mechanically, followed by three wash steps using ice-cold PBS. The metacestode vesicle pellet was then resuspended in 50 mM HEPES pH 7.4, 150 mM NaCl, 25 μg/mL digitonin supplemented with Halt Protease Inhibitor Cocktail and incubated at 4 °C for 20 min with gentle agitation. After incubation, the suspension was centrifuged at 400 × g for 10 min at 4 °C, the supernatant was transferred to a new tube, followed by another centrifugation at 18,000 × g for 20 min at 4 °C, where the supernatant then represented the cytosolic fraction. Protein concentration in the cytosolic fraction was measured using the Pierce BCA Protein Assay kit. Purity of the cytosolic fractions was checked by Western blotting, essentially as described for GL cells, by probing for the mitochondrial marker protein ATPB using a monoclonal anti-ATPB antibody diluted 1:1,000 (3D5, Abcam) and the cytosolic marker protein GAPDH using a monoclonal anti-GAPDH antibody diluted 1:2,000 (GA1R, Fisher Scientific). Whole cell lysate of *E. multilocularis* metacestode vesicles generated by RIPA extraction^72^ was used as a control including mitochondrial proteins. *Em*cMDH activity in the cytosolic fraction was monitored via the fluorescence of NADH at 460 nm at 25 °C with 150 μM of both cosubstrates and 2.5 μg cytosolic fraction per well in 50 mM Tris-HCl pH 8.0 and technical triplicates. Gossypol was added to the cytosolic fraction to a final concentration of 20 μM and activity was calculated relative to the solvent control DMSO. This experiment was done in biological triplicates with technical triplicates. Normality was confirmed using the Shapiro–Wilk test. Pairwise comparisons were performed using Welch’s *t* test. *P*-values <0.05 were considered statistically significant.

### Assessment of Metacestode Vesicle Viability

Viability of metacestode vesicles was measured essentially as described.^69^ Briefly, metacestode vesicles older than one year were purified, suspended in DMEM without phenol red, without FBS but supplemented with penicillin (100 U/mL), streptomycin (100 μg/mL) in a 1:2 ratio and distributed into 24-well plates with 2 mL of this parasite suspension per well. Metacestode vesicles were treated with 20 μM gossypol or respective amounts of the solvent control DMSO and let to incubate at 37 °C under a humid, 5% CO_2_ atmosphere for 5 days. Viability was assessed using the CellTiter-Glo assay (Promega, Dübendorf, Switzerland) according to the manufacturer’s protocol. Viability was calculated as a relative of the solvent control DMSO. This experiment was done in biological triplicates. Normality was confirmed using the Shapiro–Wilk test. Pairwise comparisons were performed using Welch’s *t* test. *P*-values <0.05 were considered statistically significant.

### Assessment of GL Cell Viability

Viability assessment of GL cells was done as previously described.^69^ Gossypol was tested 1:3 serial dilution from 120 to 0.02 μM. GL cells were incubated with the compounds at 37 °C under a humid, microaerobic (85% N_2_, 10% CO_2_, and 5% O_2_) atmosphere for 5 days. Viability was assessed using an adapted CellTiter-Glo assay (Promega, Dübendorf, Switzerland). Viability was calculated as a relative of the solvent control DMSO. IC_50_ values were determined by fitting dose–response curves from three independent experiments, each performed with three technical replicates, to a four-parameter log–logistic model separately. The reported IC_50_ represents the mean ± SD of the three independent replicates.

### Assessment of Hepa 1-6 Cell Viability

Similarly, gossypol was assessed against Hepa 1-6 cells in a 1:2 serial dilution from 120 to 0.06 μM. 5,000 cells per well were incubated in DMEM supplemented with 10% FBS, penicillin (100 U/mL), streptomycin (100 μg/mL), and tetracycline (5 μg/mL) with the compounds at 37 °C under a humid, 5% CO_2_ atmosphere for 5 days. Viability was assessed using the CellTiter-Glo assay (Promega, Dübendorf, Switzerland) according to the manufacturer’s protocol. Viability was calculated as a relative of the solvent control DMSO. IC_50_ values were determined by fitting dose–response curves from three independent experiments, each performed with three technical replicates, to a four-parameter log–logistic model separately. The reported IC_50_ represents the mean ± SD of the three independent replicates.

### Molecular Docking

*Em*cMDH tertiary structure was predicted using AlphaFold 3.^74^ Quality of the predicted structure was validated and the model was subsequently used for molecular docking analyses using CB-Dock 2,^75^ where default auto blind docking was performed. The resulting *Em*cMDH-gossypol complex was visualized and analyzed using ChimeraX.^76^

### Manuscript Preparation

Selected linguistic improvements were made with the assistance of Claude (Fable 5) and ChatGPT (GPT-5.5). Figures were prepared using Adobe Illustrator 2026. Parts of the graphical abstract were prepared with Biorender.

## Supplementary Material



## Notes

Figure S1: bioinformatic analyses of cMDHs; Figure S2: molecular cloning of cMDHs; Figure S3: cytosolic fractions of *E. multilocularis* metacestode vesicles; Figure S4: tertiary structure prediction of EmcMDH (EmuJ_000417100); Table S1: comparison of EmcMDH kinetics with various cMDHs from different species; Table S2: oligonucleotides used in this study (PDF)

## Abbreviations

AE: Alveolar Echinococcosis
LDH: lactate dehydrogenase
MD: malate dismutation
MDH: malate dehydrogenase
GL: germinal layer
