# Supplementary material for: Identification and Characterization of a Cytosolic Malate Dehydrogenase as a Potential Drug Target in Echinococcus multilocularis
Source: ACS Omega. 2026 Oct 26;11(44):68397–410. doi: 10.1021/acsomega.6c05466 (PMC13756719; doi:10.1021/acsomega.6c05466)
Supplement: Supplementary file 1 [file ao6c05466_si_001.pdf]

Identification and characterization of a cytosolic malate dehydrogenase as a potential drug target in *Echinococcus multilocularis*

Pascal Zumstein[1,2], Laura Vetter[1], María Eugenia Ancarola [1], Trix Zumkehr[1], Britta Lundström-Stadelmann*[1,3]

[1]Institute of Parasitology, Department of Infectious Diseases and Pathobiology, University of Bern, Switzerland.

[2]Graduate School for Cellular and Biomedical Sciences (GCB), University of Bern, Switzerland.

[3]Multidisciplinary Center for Infectious Diseases, University of Bern, Switzerland.

Supporting Information

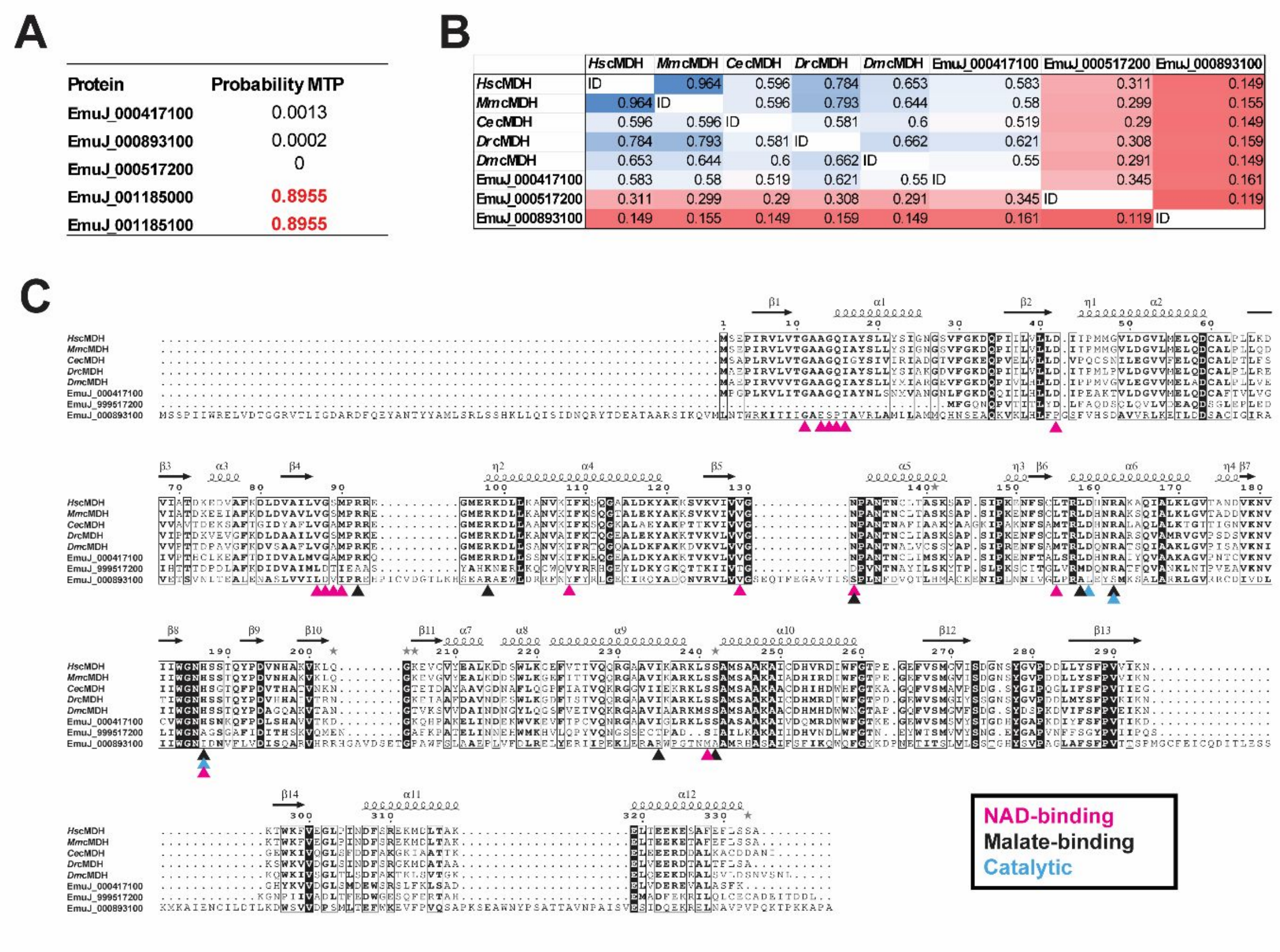

Figure S1. Bioinformatic analyses of cMDHs. (A) Predicted probability of mitochondrial transfer peptide (MTP) presence in identified MDH homologs of *E. multilocularis* (high probability values are highlighted in red). (B) cMDH homology matrix of putative cMDH homologs in *E. multilocularis* (EmuJ_000417100, EmuJ_000517200, EmuJ_000893100) and metazoan reference species (*Ce: C. elegans* (Q9UAV5), *Dm: D. melanogaster* (Q9VKX2), *Dr: D. rerio* (Q801E6), *Mm: M. musculus* (P14152), *Hs: H. sapiens* (P40925)). Dark blue indicates high sequence identity, while dark red indicates low sequence identity. (C) Multiple sequence alignment of cMDH homologs in *E. multilocularis* (EmuJ_000417100, EmuJ_000517200, EmuJ_000893100) and metazoan reference species (*Ce: C. elegans* (Q9UAV5), *Dm: D. melanogaster* (Q9VKX2), *Dr: D. rerio* (Q801E6), *Mm: M. musculus* (P14152), *Hs: H. sapiens* (P40925)). Secondary structure elements are shown above the alignment, with arrows indicating β-strands and coils representing α-helices and η-turns, secondary structure information was retrieved from *Hs*cMDH (PDB: 7RM9). Conserved residues are highlighted with black shading, while similar residues are shown in boxed regions. NAD-binding, malate-binding and catalytic residues are highlighted with arrows as shown (in purple, black and light blue, respectively), according to (Nava et al., 2011).

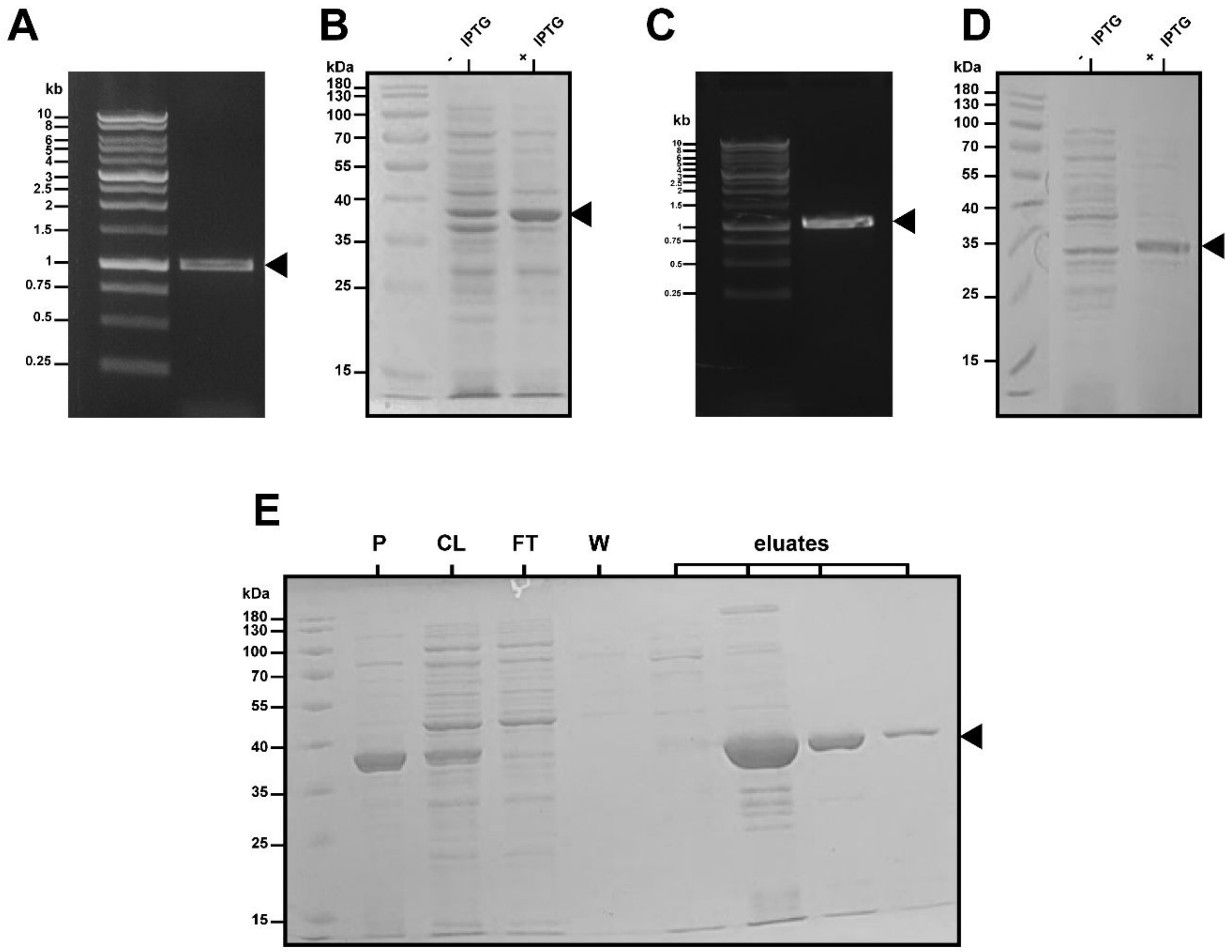

Figure S2. Molecular cloning of cMDHs. (A) *Emcmdh* amplified from *E. multilocularis* cDNA. Arrow indicates amplicon at the expected size. (B) Expression of *Em*cMDH in BL21 *E. coli*. IPTG: Isopropyl-β-D-thiogalactopyranosid. Arrow indicates protein band at the expected size of 39 kDa. (C) *Mmcmdh* amplified from Hepa 1-6 cDNA. Arrow indicates amplicon at the expected size. (D) Expression of *Mm*cMDH in BL21 *E. coli*. IPTG: Isopropyl-β-D-thiogalactopyranosid. Arrow indicates protein band at the expected size. (E) Affinity purification of *Mm*cMDH. P: pellet, CL: clarified lysate, FT: flow-through, W: wash. Arrow indicates protein band at the expected size of 39 kDa.

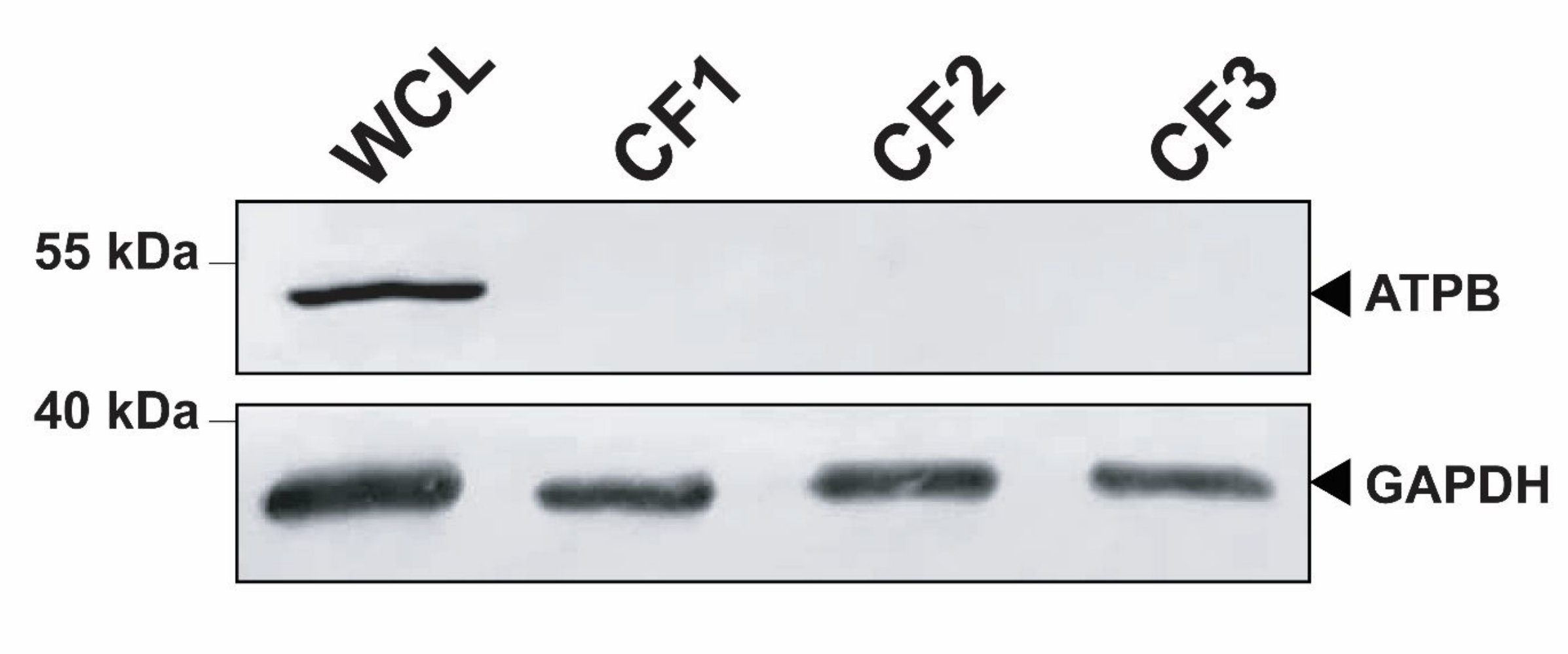

Figure S3. Cytosolic fractions of *E. multilocularis* metacestode vesicles. Western blot showing purity of cytosolic fractions. 5 µg of total proteins was loaded per lane and the membrane was probed for ATPB (mitochondrial marker) and GAPDH (cytosolic marker). WCL: whole cell lysate, CF1-3: cytosolic fractions 1-3.

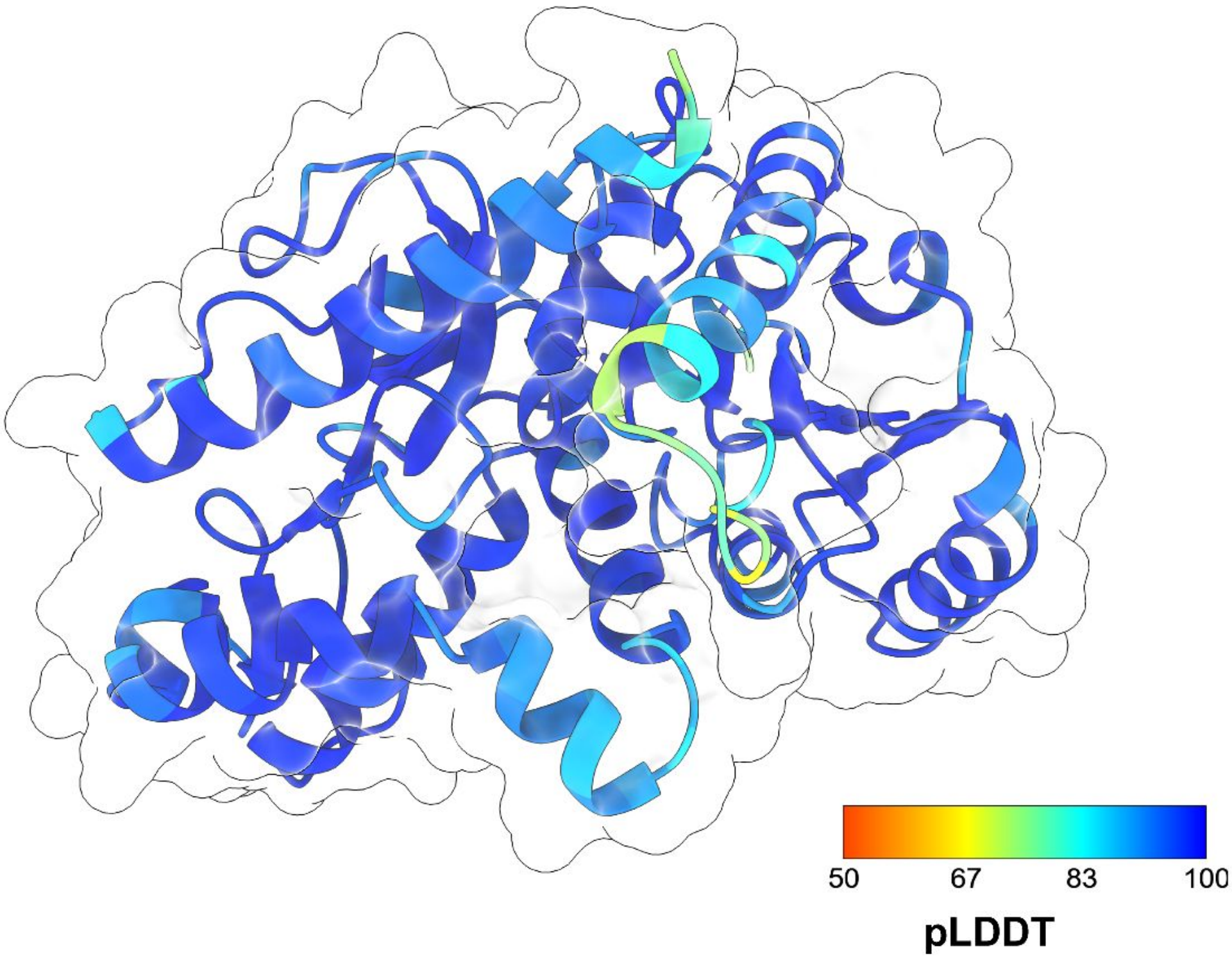

Figure S4. Tertiary structure prediction of *Em*cMDH (EmuJ_000417100). Structure was predicted using AlphaFold 3. Model is colored according to the pLDDT score as shown.

Organism	K_m (malate)	K_m (NAD^+)	K_m (oxaloacetate	K_m (NADH)
E. multilocularis	0.21 ± 0.10	0.43 ± 0.11	0.0164 ± 0.0032	0.0115 ± 0.0039
Fasciola gigantica	0.447 ± 0.047	0.309 ± 0.015	0.276 ± 0.092	0.141 ± 0.054
Spirometra mansoni	1.1933	1.9489	63.53	1.7505
Clonorchis sinensis	0.056 ± 0.006	0.094 ± 0.013	0.0096 ± 0.0009	0.007 ± 0.0004
Taenia solium	0.1	ND	0.044	ND
Sus scrofa	1.77	0.049	0.032	0.0025
Homo sapiens	ND	ND	0.00041	ND

Table S1. Comparison of *Em*cMDH kinetics with various cMDHs of different species.

Primers used for qPCR experiments

Gene	FW seq 5'-3'	RV seq 5'-3'	Efficiency
emelp	AAC ACG ATG CAA AGC ACA AGT CCC	CCT CAC ATT TGG TAT CAC CTC AAG TTC C	1.91
EmuJ_000417100	CGC TCC TTC CAT TCC GAA AGA G	CGT GTT GGG AAC TCC AGC CTT G	1.870
EmuJ_000517200	GAG TTC TGA GTG TAC TCC TGC CGA TTC	ACC ACC ATT GAC GTC CAA TAC TCA TTC	2.084
EmuJ_000893100	TTT CTT TCC CGG TCA CCA CAA GC	GGT GTC CAG AAT GCA GTT CTC AAT GG	1.939

Primers used for cloning

Gene	FW seq 5'-3'	RV seq 5'-3'
emcmdh	CAC CAT GCC TGG TCC TC	TTA CTT GAA GGA AGC AAG AG
mmcmdh	CAC CAT GTC TGA ACC AAT CAG	TCA CGC AGA GGA GAG AAA C

siRNAs used in this study

siRNA target	siRNA sequence (5' – 3')
emcmdh	CUCCAACGUAAAGAUCUUCAAGG
gfp	CUAUAACUCACAUAAUGUAUACA

Table S2. Oligonucleotides used in this study.
